# Residential Racial Composition and Black-White Obesity Risks: Differential Effects of Neighborhood Social and Built Environment

**DOI:** 10.3390/ijerph110100626

**Published:** 2014-01-02

**Authors:** Kelin Li, Ming Wen, Kevin A. Henry

**Affiliations:** 1 Department of Sociology, University of Utah, Salt Lake City, UT 84112, USA; E-Mail: ming.wen@soc.utah.edu; 2 Department of Epidemiology, Rutgers School of Public Health, NJ 08854, USA; E-Mail: henryk1@sph.rutgers.edu; 3 Rutgers Cancer Institute of New Jersey, Cancer Prevention and Control Program, New Brunswick, NJ 08903, USA

**Keywords:** obesity, neighborhood, racial segregation, social cohesion, built environment

## Abstract

This study investigates the association between neighborhood racial composition and adult obesity risks by race and gender, and explores whether neighborhood social and built environment mediates the observed protective or detrimental effects of racial composition on obesity risks. Cross-sectional data from the 2006 and 2008 Southeastern Pennsylvania Household Health Survey are merged with census-tract profiles from 2005–2009 American Community Survey and Geographic Information System-based built-environment data. The analytical sample includes 12,730 whites and 4,290 blacks residing in 953 census tracts. Results from multilevel analysis suggest that black concentration is associated with higher obesity risks only for white women, and this association is mediated by lower neighborhood social cohesion and socioeconomic status (SES) in black-concentrated neighborhoods. After controlling for neighborhood SES, black concentration and street connectivity are associated with lower obesity risks for white men. No association between black concentration and obesity is found for blacks. The findings point to the intersections of race and gender in neighborhood effects on obesity risks, and highlight the importance of various aspects of neighborhood social and built environment and their complex roles in obesity prevention by socio-demographic groups.

## 1. Introduction

The alarming obesity prevalence in the United States has triggered increasing public health attention to the population distribution and contributory factors of this epidemic in an effort to prevent related health problems such as diabetes and cardiovascular diseases [1]. Almost every major segment of the US population has seen steady increase in obesity in the past decades [1], and racial disparities persist between non-Hispanic blacks (referred to as “blacks” hereafter) and non-Hispanic whites (referred to as “whites” hereafter), particularly among women. National survey data reveal that female obesity prevalence has risen from 21.2% to 30.0% among whites and from 30.2% to 54.0% among blacks from the years 1988–1994 to 2003–2004 [2]. Individual risk factors such as socioeconomic status (SES) are shown to affect black and white adults differentially [3], and are limited in explaining black–white disparities in obesity [4]. Beyond individual characteristics, a burgeoning line of research is now shifting to an ecological approach looking at community-level environmental influences on individual obesity risks and disparities by gender and race/ethnicity [5,6].

Residential racial composition emerges as a salient neighborhood factor of residents’ health and lifestyle given the projected population growth among racial/ethnic minorities and persistent residential segregation in the US. Yet the picture is far from clear in terms of how and why neighborhood-level racial/ethnic composition may play a role in contributing to individual-level obesity risks and disparities. Williams and Collins posit that racial segregation is a fundamental cause of black-white disparities in health, mostly because institutional and personal racism disproportionately places members of minority groups into concentrated disadvantages [7]. Most recently, however, another theoretical framework has been established for the protective effects of minority concentration on health, the so-called ethnic density effects [8,9]. The underlying assumption within this framework is that residing with co-ethnics may foster better social cohesion, provide more health-promoting resources, and protect minorities from discrimination and related stress. These two competing frameworks have shed light on the dynamic pillars pertaining to US communities and have both received supporting evidence across a range of health outcomes [8,10].

This complexity is also reflected in the mixed findings in empirical work examining black concentration and weight status. Although researchers have generally agreed on its significant role, black concentration has received less attention compared to other contextual factors. In many cases it is either considered as a control variable or as an indicator of socioeconomic disadvantage in the neighborhood environment. In the research that has focused on the independent effect of black concentration, two studies report that living in black neighborhoods is associated with higher obesity risks, even after adjusting for neighborhood socioeconomic characteristics [11,12], and other research in New York City finds no effect in this association [13]. These studies, unfortunately, ignore the fact that living in black neighborhoods encompasses substantially different meanings and experiences for different racial groups, limiting the insight into independent effects of race. When community racial composition and individual race/ethnicity are jointly considered, three nationwide multilevel studies reported null finding in the relation between percentage black and body mass index (BMI) among blacks [14,15,16].

The effects of neighborhood minority concentration for whites remain largely untested. It is increasingly recognized that segregation patterns in the US not only affect the minority populations, but also influence whites in meaningful and distinct ways. Community-resilient features such as socioeconomic resources and social cohesion benefit individual health regardless of individual race [17]. However, area deprivation in association with racial segregation may sort whites with lower socioeconomic status into minority and deprived neighborhoods leading to their increased health risks, including obesity. Among residents of black-concentrated neighborhoods, whites are likely to be poorer than blacks [18] and may suffer from heightened levels of relative deprivation, which can exert additional detrimental effects on health net of absolute deprivation [19]. From the perspectives of the social disorganization theory, minority concentration may strengthen social cohesion for minority residents but may erode social cohesion for white residents. For instance, evidence suggests that living with co-ethnics fosters better social cohesion [20] and that residing in black-concentrated neighborhoods protects blacks, buffering against health risks [21]. Considering these complex circumstances, it is possible that the health impact of living in minority-concentrated communities is ambivalent for minorities but detrimental for whites. Indeed, a recent study shows that the positive association between living in neighborhoods with a high Hispanic concentration (≥25%) and the odds for obesity was slightly greater for whites than for Hispanics [14]. More studies are needed to explore this pattern and to examine whether this association differs by gender.

Moreover, neighborhood built environment, defined as human-formed, developed, or structured areas [22], is well recognized as another important dimension of the community facilitating or obstructing active living. Neighborhood features such as street connectivity and presence of exercise facilities exert influence on residents’ health-promoting behaviors as well as weight status [23], yet such physical environment resources are not equally distributed in the US and their effects differ across racial groups [24,25,26]. Regarding the link between neighborhood racial composition and obesity, it remains an empirical question whether neighborhood built environment serve as mediating or suppressing pathways and whether their effects differ by socio-demographic groups.

To address these gaps in the literature, this study specifically focuses on neighborhood black concentration and its association with residents’ obesity risks and has two aims: (1) to test whether neighborhood black concentration is positively or negatively associated with obesity risks, net of individual characteristics; (2) to explore what roles neighborhood social and built-environmental attributes, particularly social cohesion, SES, street connectivity, and park access, play in the link between black concentrations and obesity risks. Because theoretical and empirical evidence suggests that neighborhood effect on individuals depend on gender, with women being more likely influenced by community social environment [27,28], we conduct analysis by specific gender and race. Looking at moderating and mediating effects pertinent to multilevel facets of racial composition would presumably advance our understanding of individual–environment interactions and the processes underlying health risks.

## 2. Methods

### 2.1. Data

The present study is based on pooled data from the 2006 and 2008 Southeastern Pennsylvania (SEPA) Household Health Survey administrated by the Public Health Management Corporation in Bucks, Chester, Delaware, Montgomery, and Philadelphia counties. This biennial cross-sectional survey drew a stratified probability sample from 54 service areas where each had about 30,000 to 75,000 adult residents, and was conducted through telephone interviews with people aged 18 and older. One eligible adult respondent was chosen from each household based on selection criteria. People aged 60 and older were over-sampled for the purpose of asking specific questions to this age group. Self-reported person-level data from the SEPA Household Health Survey were then linked to census-tract profiles obtained from 2005–2009 American Community Survey (ACS), US Census, and Geographic Information System (GIS) built-environment data. After excluding tracts with only 1 resident (*N* = 13) and respondents who had missing values on weight status or any of the predictors (*N* = 557), the final analytical sample included 12,730 whites and 4,290 blacks residing in 953 tracts, with an average of 21 residents per tract (SD: 11; range: 2–196).

### 2.2. Individual-Level Measures

The outcome variable was a dichotomous indicator of being obese if a respondent had a BMI equal to or higher than 30. BMI was calculated based on self-reported height and weight following the formula BMI = weight (kg)/height (m)^2^.

Individual covariates included self-reported race (white *vs*. black), gender (male *vs*. female), age, marital status (married/living with partner *vs*. single/separated/divorced/widowed), nativity (US born *vs*. foreign born), educational attainment (high school or below, some college, and college or above), income (below 100% federal poverty line, 100%–200% federal poverty line, and at or above 200% federal poverty line), smoking status (currently a smoker or not), and survey year (2006 *vs*. 2008). We also included an age-squared term in the models to account for curvilinear relationship between age and obesity.

### 2.3. Neighborhood-Level Measures

Percentage black was obtained from ACS 2005–2009 data and was a measure of racial composition in each census tract (ranging from 0 to 1). The original continuous measure was then dichotomized as whether a census tract had 25% or more black residents [14,29]. Racial/ethnic concentration as proxy measure of residential isolation has been often used in prior studies [6,12,30,31], and also allows the examination of health risks or benefits of living with co-ethnics or with certain groups [32].

Accounting for neighborhood SES is essential in detangling ethnic density effects from area deprivation associated with residential segregation [8,33]. We used information on tract-level percentage of college graduates, percentage of unemployed residents, percentage of residents living in poverty, and percentage of households with annual income $75,000 or above from ACS 2005–2009 data, and performed principle-component factor analysis to construct a summary scale based on the above four items (ranging from −3.20 to 1.73; alpha = 0.82).

Neighborhood social cohesion was an aggregated summary measure based on the following three questions in the SEPA Household Health Survey: (1) “Please rate how likely people in your neighborhood are willing to help their neighbors with routine activities such as picking up their trash cans, or helping to shovel snow. Would you say that most people in your neighborhood are always, often, sometimes, rarely, or never willing to help their neighbors?” (2) “Please tell me if you strongly agree, agree, disagree, or strongly disagree with the following statement: I feel that I belong and am a part of my neighborhood”. (3) “Please tell me if you strongly agree, agree, disagree or strongly disagree with the following statement: Most people in my neighborhood can be trusted”. We reversed the item responses and created a summary score of perceived social cohesion for each respondent using principle component factor analysis (alpha = 0.66), with higher value indicating more social cohesion. We then aggregated this score to each census tract based on mean response (ranging from −1.92 to 1.47).

GIS-based objective measures of neighborhood built environment consisted of street connectivity and park accessibility. Street connectivity was measured by the number of intersections per square mile in each census tract (ranging from 8.79 to 1,071.43) and spatial park accessibility was measured by weighted distance (in miles) from the neighborhood centroid to the nearest seven parks (ranging from 0.22 to 8.92) [34,35]. Finally, tract-level percentage of residents living in the same house in the year 1995 was obtained from the 2000 US Census and is used as a covariate in the analysis to capture residential stability (ranging from 0.05 to 0.92).

### 2.4. Statistical Analysis

We used multilevel random intercept logistic regression models to account for the clustering nature of the data, where individuals are nested within neighborhoods. Weighted group-specific analyses were conducted to examine contextual effects of black concentration on obesity risks separately for white women, white men, black women, and black men. In each set of the stratified analysis, we first examined the crude effect of black concentration on obesity risks while adjusting for individual-level covariates (Model 1). In Model 2, we included social cohesion, one indicator of neighborhood social environment, to assess whether it served as a pathway for black concentration. Then in Model 3, neighborhood SES was added to see whether the observed association between black concentration and obesity was attributable to area deprivation and socioeconomic disadvantage. Finally, we added the two built-environment measures of street connectivity and park accessibility as potential mediators (Model 4). Results were reported as odds ratios with 95% confidence intervals. We incorporated individual sampling weights to account for study design and sampling selection bias and performed our analyses in Stata 11.2 and Mplus 7.11.

Following multivariate regression analysis, we performed formal multilevel mediation analysis to assess the effect that each hypothesized mediator had in attenuating the association between black concentration and individual obesity. Our approach followed a single 2→2→1 mediation model, where a level-2 mediator was examined in the relation between a level-2 predictor and a level-1 outcome [36]. First, a single-level model involving only neighborhood variables was specified to obtain the coefficient between black concentration and each potential mediator (a-path). Second, a multilevel random intercept model was specified to examine each mediator and individual obesity risks (b-path), adjusting for black concentration (c’-path). Mediated effects were computed by multiplying coefficients for the a- and b-paths (β_a_*β_b_); standard errors were calculated using the first-order Taylor series expression; and the Sobel test was used to test for the significance of mediated effects.

**Table 1:** Unweighted sample characteristics.

	White Women	White Men	Black Women	Black Men
***Individual-level Measures***				
Obese	20.74%	24.06%	40.79%	31.27%
Age	54.57 (0.18)	53.65 (0.24)	49.28 (0.30)	50.52 (0.48)
Married/living with partner	58.74%	66.78%	30.33%	45.27%
US born	96.40%	96.12%	95.07%	92.35%
Educational attainment				
High school or below	37.10%	31.36%	52.50%	54.90%
Some college	19.97%	18.75%	24.73%	22.51%
College or above	42.94%	49.89%	22.78%	22.59%
Income				
<100% FPL	4.54%	3.04%	20.03%	13.14%
100%–200% FPL	13.33%	9.61%	27.29%	24.91%
≥200% FPL	82.14%	87.35%	52.69%	61.94%
Current smoker	18.21%	18.66%	25.56%	28.18%
Survey year 2008	50.78%	49.51%	51.89%	55.24%
**Sample Size (N)**	**8,224**	**4,506**	**3,126**	**1,164**
***Neighborhood-level Measures***				
Percent black ≥ 25%	20.75%	19.05%	46.29%	55.28%
Social cohesion	0.06 (0.01)	0.06 (0.01)	−0.15 (0.02)	−0.18 (0.02)
Socioeconomic status	0.15 (0.03)	0.20 (0.03)	−0.37 (0.04)	−0.52 (0.05)
Street connectivity	166.51 (4.96)	163.03 (5.06)	227.00 (6.59)	243.40 (7.63)
Park accessibility	1.59 (0.04)	1.62 (0.04)	1.16 (0.04)	1.10 (0.04)
Residential stability	0.58 (0.00)	0.58 (0.00)	0.57 (0.01)	0.57 (0.01)
**Number of Tracts**	**853**	**819**	**553**	**407**

Note: Data shown are percentage or mean (standard deviation).

## 3. Results

### 3.1. Descriptive Statistics

Unweighted descriptive statistics are presented in Table 1, stratified by race and gender. Consistent with the national trend, obesity prevalence was much lower among whites than it was among blacks, and this gap was even larger for women. The sample consisted of more women than men, and black respondents on average were younger than whites. The majority of whites were married or living with a partner, whereas the majority of blacks were not married. SES achievement gap by race was apparent in terms of education and income. Nearly half of whites had a college degree, more than twice as many as blacks. In contrast, many more blacks lived below the federal poverty line compared to only a few whites. At the neighborhood level, black concentration was noticeable as about one half of blacks’ neighborhoods were composed of 25% or more black residents, but the corresponding number was about one fifth for whites. Neighborhood SES and social cohesion were both higher among whites than blacks. But blacks seemed to enjoy more health-promoting built environments, as their neighborhoods had better street connectivity and shorter distances to parks. Bivariate associations between neighborhood variables are presented in Table 2.

**Table 2:** Correlation matrix between neighborhood-level variables.

	(1)	(2)	(3)	(4)	(5)
**(1) Percent black ≥ 25%**	1.000				
**(2) Social cohesion**	−0.512	1.000			
**(3) Socioeconomic status**	−0.635	0.657	1.000		
**(4) Street connectivity**	0.400	−0.439	−0.565	1.000	
**(5) Park accessibility**	−0.365	0.376	0.404	−0.495	1.000
**(6) Residential stability**	−0.045	0.128	0.002	−0.130	0.059

### 3.2. Multivariate Regression Analysis

Table 3 presents results from the multilevel random intercept logistic regression models for whites. Among white women, Model 1 indicates that black concentration was associated with higher odds of being obesity (OR = 1.43, *p* < 0.001). But this association became weaker (OR = 1.28, *p* < 0.05) when neighborhood social cohesion was added in Model 2 (OR = 0.77, *p* < 0.05). In Model 3, this association seemed to be explained away by area deprivation as adding neighborhood SES rendered the effect of black concentration insignificant. Neighborhood SES was a significant predictor that was negatively associated with obesity risks (OR = 0.74, *p* < 0.001). In Models 4, none of the neighborhood built-environmental variables were significant for white women.

The second part of Table 3 shows model estimates for white men. Model 1 shows no significant relationship of black concentration and odds of obesity for white men, but neighborhood social cohesion was a significant and negative correlate of obesity in Model 2 (OR = 0.67, *p* < 0.01). Black concentration was negatively linked to obesity (OR = 0.64, *p* < 0.05) only when neighborhood SES was controlled for, which was a negative predictor itself (OR = 0.75, *p* < 0.001) (Model 3). The effect of black concentration remained stable when the two built-environmental measures were added in Model 4. Notably, street connectivity was significantly and negatively associated with obesity among white men (OR = 0.99, *p* < 0.01). The analyses did not show significant neighborhood effects for blacks (Table 4).

### 3.3. Mediation Analysis

**Figure 1:**
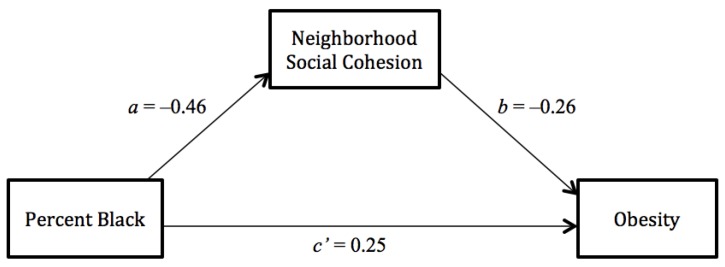
Path diagram depicting neighborhood social cohesion as the mediator between black concentration and obesity among white women.

**Figure 2:**
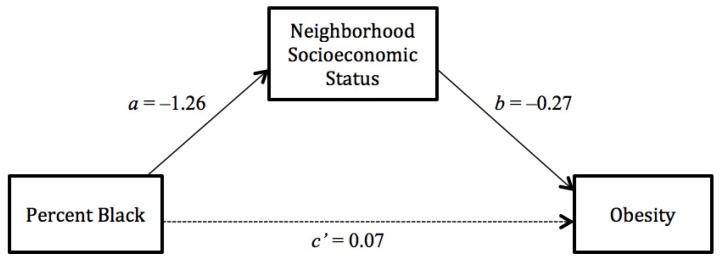
Path diagram depicting neighborhood SES as the mediator between black concentration and obesity among white women.

Based on the patterns shown in the multivariate regression, we performed formal multilevel mediation analysis among white women to assess the mediated effect of each hypothesized neighborhood-level mediator. Figure 1 and Figure 2 present results from single mediation models for neighborhood social cohesion and neighborhood SES, respectively.

**Table 3:** Multilevel logistic regression odds ratio predicting obesity for white women and men.

	White Women (*N* = 8,224)				White Men (*N* = 4,506)			
	Model 1	Model 2	Model 3	Model 4	Model 1	Model 2	Model 3	Model 4
***Neighborhood-level Measures***								
Percent black ≥ 25%	1.43 ***	1.28 *	1.09	1.09	0.89	0.74 ^+^	0.64 *	0.65 *
	[1.17–1.75]	[1.02–1.60]	[0.86–1.36]	[0.87–1.38]	[0.66–1.20]	[0.53–1.03]	[0.45–0.92]	[0.46–0.93]
Social cohesion		0.77 *	1.11	1.08		0.67 **	0.93	0.89
		[0.62–0.97]	[0.86–1.44]	[0.83–1.41]		[0.50–0.89]	[0.66–1.29]	[0.63–1.24]
Socioeconomic status			0.74 ***	0.72 ***			0.75 ***	0.69 ***
			[0.66–0.83]	[0.64–0.81]			[0.64–0.88]	[0.58–0.81]
Street connectivity				1.00				0.99 **
				[1.00–1.00]				[0.99–1.00]
Park accessibility				1.02				0.99
				[0.96–1.09]				[0.91–1.08]
Residential stability	1.33	1.48	1.18	1.12	1.90	2.25 ^+^	1.83	1.41
	[0.64–2.75]	[0.71–3.1]	[0.57–2.40]	[0.53–2.33]	[0.76–4.77]	[0.88–5.73]	[0.72–4.67]	[0.54–3.66]
***Individual-level Measures***								
Age	1.11 ***	1.11 ***	1.11 ***	1.11 ***	1.14 ***	1.14 ***	1.14 ***	1.14 ***
	[1.08–1.14]	[1.08–1.14]	[1.08–1.14]	[1.08–1.14]	[1.10–1.18]	[1.10–1.18]	[1.10–1.18]	[1.10–1.18]
Ages quared	1.00 ***	1.00 ***	1.00 ***	1.00 ***	1.00 ***	1.00 ***	1.00 ***	1.00 ***
	[1.00–1.00]	[1.00– 1.00]	[1.00–1.00]	[1.00–1.00]	[1.00–1.00]	[1.00–1.00]	[1.00–1.00]	[1.00–1.00]
Married	0.69 ***	0.70 ***	0.71 ***	0.70 ***	1.03	0.97	0.99	0.98
	[0.60–0.79]	[0.60–0.80]	[0.61–0.81]	[0.61–0.81]	[0.92–1.16]	[0.80–1.17]	[0.82–1.20]	[0.81–1.18]
US born	1.72 **	1.73 **	1.71 **	1.71 **	1.10	1.11	1.12	1.13
	[1.20–2.48]	[1.20–2.50]	[1.19–2.48]	[1.18–2.47]	[0.72–1.68]	[0.73–1.70]	[0.73–1.72]	[0.74–1.72]
Education ^a^	0.73 ***	0.74 ***	0.77 ***	0.77 ***	0.79 ***	0.80 ***	0.83 ***	0.84 ***
	[0.67–0.79]	[0.68– 0.80]	[0.70–0.83]	[0.70–0.84]	[0.72–0.87]	[0.73–0.88]	[0.75–0.92]	[0.76–0.92]
Income ^b^	0.77 ***	0.78 ***	0.80 **	0.80 **	0.90	0.92	0.95	0.93
	[0.68–0.87]	[0.69–0.89]	[0.70–0.91]	[0.70–0.91]	[0.75–1.07]	[0.77–1.10]	[0.79–1.13]	[0.78–1.12]
***Individual-level Measures***								
Current smoker	0.69 ***	0.68 ***	0.68 ***	0.68 ***	0.73 **	0.72 **	0.71 **	0.71 **
	[0.57–0.83]	[0.57–0.82]	[0.56–0.82]	[0.56–0.82]	[0.58–0.91]	[0.58–0.91]	[0.57–0.89]	[0.57–0.89]
Year 2008	1.00	1.00	0.99	0.99	1.19 *	1.18 *	1.18 *	1.18 *
	[0.88–1.13]	[0.88–1.13]	[0.87–1.12]	[0.87–1.12]	[1.01–1.39]	[1.00–1.38]	[1.01–1.39]	[1.00–1.38]
**Level 2 variance**	0.20 ***	0.20 ***	0.17 ***	0.17 ***	0.35 ***	0.33 ***	0.30 ***	0.28 ***
**Intraclass correlation**	0.06	0.06	0.05	0.05	0.11	0.10	0.09	0.08
**AIC**	7,881.35	7,877.96	7,855.43	7,857.40	4,828.89	4,822.07	4,810.86	4,804.41
**BIC**	7,965.53	7,969.15	7,953.64	7,969.64	4,905.85	4,905.44	4,900.65	4,907.02

Note: 95% Confidence Intervals are in parentheses; ^a^ Education is treated as a continuous variable in the models. It has three levels: “high school or below”, “some college”, “college or above”; ^b^ Income is treated as a continuous variable in the models. It has three levels: “below 100% FPL”, “100–200% FPL”, “at or above 200% FPL”; *** *p* < 0.001, ** *p* < 0.01, * *p* < 0.05, ^+^ *p* < 0.10 (two-tailed test).

**Table 4:** Multilevel logistic regression odds ratio predicting obesity for black women and men.

	Black Women ( *N* = 3,126)				Black Men ( *N* = 1,164)			
	Model 1	Model 2	Model 3	Model 4	Model 1	Model 2	Model 3	Model 4
***Neighborhood-level Measures***								
Percent black ≥ 25%	1.10	1.03	0.95	0.93	0.90	0.87	0.87	0.95
	[0.89–1.37]	[0.76–1.29]	[0.74–1.22]	[0.72–1.20]	[0.64–1.27]	[0.60–1.25]	[0.59–1.28]	[0.63–1.43]
Social cohesion		0.77 ^+^	0.92	0.93		0.92	0.91	0.87
		[0.59–1.00]	[0.66–1.27]	[0.67–1.29]		[0.60–1.41]	[0.53–1.56]	[0.51–1.50]
Socioeconomic status			0.88 ^+^	0.90			1.01	1.01
			[0.76–1.01]	[0.77–1.05]			[0.79–1.28]	[0.78–1.29]
Street connectivity				1.00				1.00
				[1.00–1.00]				[1.00–1.00]
Park accessibility				0.93				1.21
				[0.77–1.13]				[0.95–1.56]
Residential stability	0.47 ^+^	0.53	0.44 ^+^	0.45 ^+^	1.42	1.51	1.53	1.32
	[0.20–1.10]	[0.23–1.24]	[0.18–1.08]	[0.19–1.16]	[0.37–5.44]	[0.40–5.72]	[0.41–5.77]	[0.35–5.00]
***Individual-level Measures***								
Age	1.14 ***	1.14 ***	1.14 ***	1.14 ***	1.17 ***	1.17 ***	1.17 ***	1.17 ***
	[1.10–1.17]	[1.10–1.17]	[1.10–1.17]	[1.10–1.17]	[1.11–1.24]	[1.11–1.24]	[1.11–1.24]	[1.11–1.24]
Age squared	1.00 ***	1.00 ***	1.00 ***	1.00 ***	1.00 ***	1.00 ***	1.00 ***	1.00 ***
	[1.00–1.00]	[1.00–1.00]	[1.00–1.00]	[1.00–1.00]	[1.00–1.00]	[1.00–1.00]	[1.00–1.00]	[1.00–1.00]
Married	0.79 *	0.79 *	0.79 *	0.80 *	0.90	1.07	1.07	1.08
	[0.66–0.96]	[0.66–0.96]	[0.66–0.96]	[0.66–0.97]	[0.75–1.08]	[0.79–1.46]	[0.78–1.46]	[0.79–1.47]
US born	1.86 **	1.84 **	1.82 **	1.81 **	2.52 **	2.47 **	2.48 **	2.48 **
	[1.25–2.76]	[1.24–2.74]	[1.22–2.71]	[1.21–2.69]	[1.30–4.89]	[1.28–4.78]	[1.28–4.80]	[1.28–4.78]
Education ^a^	0.78 ***	0.79 ***	0.80 ***	0.80 ***	0.85 ^+^	0.86 ^+^	0.86 ^+^	0.86 ^+^
	[0.69–0.87]	[0.71–0.88]	[0.71–0.89]	[0.71–0.90]	[0.71–1.02]	[0.72–1.03]	[0.71–1.03]	[0.72–1.03]
Income ^b^	0.80 ***	0.81 ***	0.81 **	0.81 **	1.22 ^+^	1.24 ^+^	1.24 ^+^	1.23 ^+^
	[0.71–0.90]	[0.71–0.91]	[0.72–0.91]	[0.72–0.92]	[0.97–1.53]	[0.99–1.55]	[0.99–1.56]	[0.99–1.55]
Current smoker	0.67 ***	0.67 ***	0.67 ***	0.66 ***	0.55 **	0.55 ***	0.55 **	0.55 **
	[0.55–0.82]	[0.55–0.82]	[0.55–0.81]	[0.54–0.81]	[0.39–0.77]	[0.39–0.77]	[0.39–0.77]	[0.39–0.78]
Year 2008	0.91	0.91	0.92	0.92	1.02	1.03	1.03	1.01
	[0.77–1.08]	[0.77–1.09]	[0.77–1.09]	[0.77–1.09]	[0.76–1.37]	[0.77–1.38]	[0.77–1.38]	[0.76–1.36]
**Level 2 variance**	0.17 ***	0.17 **	0.18 **	0.17 **	0.22	0.22	0.22	0.19
**Intraclass correlation**	0.05	0.05	0.05	0.05	0.07	0.07	0.07	0.06
**AIC**	4,042.83	4,041.40	4,039.42	4,042.17	1,376.37	1,378.25	1,380.25	1,381.66
**BIC**	4,115.40	4,120.02	4,124.08	4,138.93	1,437.09	1,444.02	1,451.08	1,462.62

Note: 95% Confidence Intervals are in parentheses; ^a^ Education is treated as a continuous variable in the models. It has three levels: “high school or below”, “some college”, “college or above”; ^b^ Income is treated as a continuous variable in the models. It has three levels: “below 100% FPL”, “100–200% FPL”, “at or above 200% FPL”; *** *p* < 0.001, ** *p* < 0.01, * *p* < 0.05, ^+^ *p* < 0.10 (two-tailed test).

Figure 1 shows that black concentration was significantly associated with neighborhood social cohesion (β_a_ = −0.46, SE = 0.03, *p* < 0.001), which was significantly associated with obesity itself (β_b_ = −0.26, SE = 0.11, *p* < 0.05). The association between black concentration and obesity remained significant even when the mediator of social cohesion was controlled for (β_c’_ = 0.25, SE = 0.11, *p* < 0.05). The mediated effect of social cohesion was also statistically significant (β_a_*β_b_ = 0.12, SE = 0.05, *p* < 0.05).

In Figure 2, black concentration was significantly associated with neighborhood SES (β_a_ = −1.26, SE = 0.06, *p* < 0.001), and neighborhood SES was also significantly associated with obesity (β_b_ = −0.27, SE = 0.05, *p* < 0.001). However, the association between black concentration and obesity was no longer significant when neighborhood SES was added as a mediator (β_c’_ = 0.07, SE = 0.11, *p* = 0.57). The mediated effect of neighborhood SES was statistically significant (β_a_*β_b_ = 0.34, SE = 0.07, *p* < 0.001).

## 4. Discussion

The main purposes of this multilevel study were to examine whether neighborhood black concentration and residents’ obesity risk were linked for four demographic groups defined by gender and black/white race and to explore whether these associations were attributable to neighborhood social and built-environmental features. The results revealed complex patterns at the intersections of race and gender in obesity risks. As expected, black concentration was associated with higher odds of obesity for white women, and this association was mediated by lower level of social cohesion and socioeconomic status in black-concentrated neighborhoods. Among white men and blacks, there was no significant association between black concentration and obesity risks. But white men in black-concentrated neighborhoods were shown to have lower odds of obesity after adjusting for neighborhood SES.

To date, some research focusing on black residential concentration and weight status has found that increasing proportion of black residents in a neighborhood was associated with higher obesity risks without differentiating individual race and gender [11,12]. This study used recent data and extended previous studies by looking at race and gender groups separately. Our results suggest that the observed detrimental effects of black concentration largely apply to white women, and community-level SES profiles and social cohesion play mediating roles between black concentration and obesity risks. These patterns are consistent with social disorganization theory in that area deprivation and the breakdown of public order as a result of ethnic heterogeneity may increase obesity risks, but only among white women. After controlling for neighborhood SES, the negative association of black concentration and obesity among white men is unexpected. It is possible that white men living in high density black neighborhoods are more socioeconomically disadvantaged than blacks, therefore they may be more likely to have manual labor jobs that entail heavy work-related physical activity, or to take public transportation because they do not own a car. Both forces could lead to greater energy consumption among white men compared to their black neighbors. For blacks, the findings do not support either ethnic density effects hypothesis or the argument that segregation is a fundamental cause of black-white health disparities, and echoes previous studies that reported null findings in the association between black concentration and obesity risks [14,15,16]. It is possible that both protective and detrimental influences on obesity risks operate together in black neighborhoods and lead to this null association, pointing to an arena of research to further explore the dynamic mechanisms underlying this association in order to reduce high obesity prevalence among blacks more efficiently.

It is important to note that the detrimental effects of black concentration for white women should not be interpreted as an argument for the status quo of residential segregation across US communities. Although minority concentration, or proportion of residents belonging to a racial/ethnic group in a neighborhood, has long been used as a proxy measure of racial/ethnic segregation, it entails considerably different meanings distinct from formal segregation measures such as dissimilarity and isolation indices [27]. While segregation indices reflect processes and dynamics of racial inequality and potential interaction at the societal level [32], measures of racial/ethnic concentration may better capture the essence of ethnic density effects. Such distinctions are also reflected in empirical work examining mortality risks, as recent syntheses suggest that using these two types of measures of residential settlement patterns by race/ethnicity could have contradictory findings [32]. There is emerging scholarly interest to jointly consider these two measures [37,38], which could be an important new direction for future research. It is possible that the underpinnings behind residential segregation and racial composition operate together to shape their health implications in the US.

Another unique contribution of this study is testing several hypothesized pathways linking residential racial composition and obesity risks, which has been scarce in existing literature. Neighborhood socioeconomic environment is consistently shown to influence individual health including obesity, above and beyond individual risk factors [5,39]. Our finding that neighborhood SES was negatively associated with obesity risks is consistent with this literature with the exception of blacks, for whom no neighborhood effect was observed. Whether neighborhood SES plays a mediating role for minority concentration has not been well examined, particularly with different social groups. One study in Utah reports that neighborhood SES partially mediated the effect of Latino concentration on obesity [6], while another national study found little mediating effect for black and Mexican Americans [28]. In this study, while neighborhood SES played a mediating role for white women, it amplified the protective effects of black concentration for white men. Adequately adjusting for area deprivation and concentrated disadvantage related to minority concentration remains a key consideration in researching segregation and health.

We have also considered perceived neighborhood social cohesion in our analyses. Community-level social cohesion protects residents from a range of physical and psychological health risks [21,40], and our finding that higher social cohesion score is associated with lower odds of obesity among whites is in line with these studies. The results also indicate that neighborhood social cohesion mediates the effects of black concentration among white women, and this mediated effect is further attributable to neighborhood SES. For blacks, the current study did not find any main effect of social cohesion on obesity risks. There are many other aspects of community social environment that we were not able to include in this study, such as racial discrimination and neighborhood safety. Given that neighborhood social environment affects obesity-related risks [41], more work on the potential roles of different aspects of social environment is warranted.

With regard to the built environment, we show that neighborhood street connectivity had a small but significant protective effect against obesity among white men. Such findings underscore the salience of community physical environment that is favorable to obesity-preventing behaviors such as physical activity [27,42]. Interestingly, it was street connectivity rather than park accessibility that had effects for white men. Because street connectivity is a proxy indicator of neighborhood walkability, it is likely that white men living in black-concentrated neighborhoods, compared to their black counterparts, are more engaged in total physical activity for transportation and occupational purposes because they tend to be more socioeconomically disadvantaged. For example, evidence has shown that residents in disadvantaged neighborhoods were more likely to walk than those in advantageous neighborhoods, despite their concern of neighborhood safety [43]. This finding suggests that the salutary built-environmental features available in one’s neighborhoods may have not been taken advantage of among other groups. Such social disparities in use of neighborhood built-environmental resources have drawn recent attention despite the continuing endeavor to call for health-promoting amenities in community design [25,44].

This study is not without limitations. First, the cross-sectional design is limited in handling estimation bias as a result of the nonrandom nature of individuals’ neighborhood choice, thus disallowing any causal inference of contextual influences on obesity risks. The selection bias may vary systematically across racial groups leading to differential effects of residential segregation for different groups. Second, our individual-level measures are based on self-reported responses. It is likely that group-specific bias and recall bias on key measures such as BMI and social cohesion would over- or underestimate group differences. In addition, because this study is based on a sample of whites and blacks collected in the Southeastern Pennsylvania area, generalization of the findings should always be done with caution.

## 5. Conclusions

This study provides new evidence on the associations between neighborhood social (*i.e.*, black concentration, social cohesion and neighborhood SES) and built (*i.e.*, street connectivity and park spatial accessibility) environmental features and individual obesity risks, highlighting the importance of considering the intersection of race and gender in neighborhood effects on obesity research. While we find that living in high density black neighborhoods can be a significant correlate of obesity risks, conducting stratified analyses by race and gender is helpful for revealing more nuanced and complex patterns. To our knowledge, this study is among the first to investigate differential pathways underlying the link between racial concentration and obesity risks, particularly for whites. They also point to the need to further examine the roles of residential segregation and minority concentration in contributing to individuals’ obesity risk net of individual background. Perhaps a fruitful elaboration of the current study would be to explore whether residential patterns by race/ethnicity matter to lifestyles factors that are immediately relevant for energy balance, namely physical activity and diet. More evidence is warranted on the mechanisms underlying these observed associations to make policy recommendations tailored for group-specific needs to thus be more effective than a general approach.
